# Effectiveness of a practical multi-setting lifestyle intervention on the main BMI trajectories from childhood to young adulthood: A community-based trial

**DOI:** 10.1186/s12889-022-14306-2

**Published:** 2022-10-31

**Authors:** Parnian Parvin, Hasti Masihay-Akbar, Leila Cheraghi, Soha Razmjouei, Amirali Zareie Shab-khaneh, Fereidoun Azizi, Parisa Amiri

**Affiliations:** 1 Research Center for Social Determinants of Health, Research Institute for Endocrine Sciences, Shahid Beheshti University of Medical Sciences, Tehran, Iran; 2 Department of Epidemiology and Biostatistics, Research Institute for Endocrine Sciences, Shahid Beheshti University of Medical Sciences, Tehran, Iran; 3 Endocrine Research Center, Research Institute for Endocrine Sciences, Shahid Beheshti University of Medical Sciences, Tehran, Iran

**Keywords:** Lifestyle, Behaviors, Intervention, Health promotion, BMI trajectories, Lifespan

## Abstract

**Background:**

Preventing overweight in childhood and subsequent stages of life is still a global challenge. Despite numerous relevant lifestyle interventions, data on their impact on different BMI change pathways over time is rare. The present study aimed to investigate the effect of a multi-setting lifestyle intervention on BMI trajectories from childhood to young adulthood.

**Methods:**

A multi-setting lifestyle intervention at the school, family, and community levels have been conducted in the Tehran Lipid and Glucose Study framework. A total of 2145 children (4–18 years, 49% boys, and 18% intervention) were recruited for the baseline assessment and were followed through five follow-up examinations during a median of 16.1 years. Using a group-based trajectory model, BMI trajectories from childhood to young adulthood were identified, and their association with the implemented intervention was assessed.

**Results:**

Four trajectory groups of BMI from childhood to young adulthood were identified, including *Normal weight* (41%), *Young adulthood overweight* (36%), *Early childhood increasing overweight and adulthood obesity* (19%), and *Early childhood increasing obesity* (4%). Only *Young adulthood overweight* and *Early childhood increasing obesity* were affected by the intervention and were concomitant with lower BMI levels than the control group, with the highest estimated effect in the latter (β=-0.52 and p = 0.018; β=-1.48 and p < 0.001, respectively).

**Conclusion:**

The current findings indicate the highest effectiveness of a practical, healthy lifestyle intervention on those whose obesity started in the early years of life or youth. Our results could help policymakers and planners design more targeted lifestyle modification and weight control interventions.

**Trial registration:**

This study is registered at Iran Registry for Clinical Trials, a WHO primary registry (http://irct.ir). The Iran Registry for Clinical Trials ID and date are IRCTID:IRCT138705301058N1, 29/10/2008.

**Supplementary Information:**

The online version contains supplementary material available at 10.1186/s12889-022-14306-2.

## Background

As one of the most critical public health challenges of the century, childhood obesity is the underlying reason for many chronic diseases and excessive weight gain in adulthood [1, 2]. Obesity prevalence has increased in children and adolescents and reached 23.8% and 22.6% in developed and 12.9% and 16.2% in developing countries for boys and girls, respectively [3]. In Iran, based on a recent systematic review and meta-analysis, childhood obesity was estimated from 11 to 24% among school-aged children, slightly higher in boys than girls [4]. Beyond physical complications, it is proven that obesity can negatively affect children’s emotional health and health-related quality of life [5], which shows the necessity of intervention in this field.

Among the various individual and socio-environmental factors that determine weight status during the developmental period of childhood and adolescence, an unhealthy lifestyle, mainly characterized by a high-calorie diet and low physical activity, is the main factor involved [6]. Accordingly, several national surveys in Iran revealed a high prevalence of inactivity, alarming daily fast food consumption rates, and insufficient intake of fresh fruits and vegetables among children [7, 8]. Based on the available evidence, home, school, and community are the three main settings for promoting healthy lifestyles in early life [9]. Hence, long-term multi-setting interventions encompassing all of the above environments were the most successful programs in improving weight status and related behaviors in childhood [10–12].

During the last decades, several cross-sectional and short-term longitudinal studies, mostly in developed countries, assessed the effectiveness of lifestyle interventions on the weight management process during childhood [13, 14]. These studies focused on the average BMI change without determining the developmental sub-groups of BMI as the latent layers of this phenomenon within the target populations. Meanwhile, existing evidence revealed different impacts of behavioral interventions on various BMI sub-groups. However, knowledge about the effectiveness of lifestyle interventions on body weight progression from childhood to adulthood is sparse. Considering the outcome index as the average BMI, regardless of related sub-groups, may lead to the loss of valuable information regarding the impact of these interventions on latent patterns of weight change over time and incorrect judgments about program efficacy. In the present study -with the hypothesis that lifestyle intervention effects may differ on various weight trends- for the first time, we aimed to investigate the impact of a multi-setting lifestyle intervention on BMI trajectories from the early years of life to young adulthood from 2001 to 2018.

## Methods

### Study design

The current study uses the Tehran Lipid and Glucose Study (TLGS) data. TLGS is an ongoing large-scale, community-based prospective study performed on a representative sample of residents of district-13 of Tehran, the capital of Iran. The mentioned district was chosen mainly because city-level data showed a high stability rate in that region. Also, the age distribution in the section represented the entire Tehran population. The main goal of this cohort is to prevent non-communicable disorders (NCDs) by creating a healthy lifestyle and reducing the risk factors of NCDs.

The TLGS consists of two main junctures: first, a cross-sectional baseline assessment to investigate the prevalence of NCDs and relevant risk factors (1999–2001), and second, the subsequent follow-up study accompanied by lifestyle intervention. The multistage cluster random sampling method was used to recruit the participants. In stage one, three of the 20 health care centers of district-13 of Tehran were selected. In the second stage, data of 15,005 residents (aged ≥ 3 years) were randomly collected from those health care centers. The TLGS intervention was initiated after the baseline data acquisition. One of the health centers which was far from the other two was selected as the intervention center [15, 16]. Participants were invited for a re-exam every three years; five data recollections were conducted from 2001 to 2018.

### Study participants

**Fig. 1:**
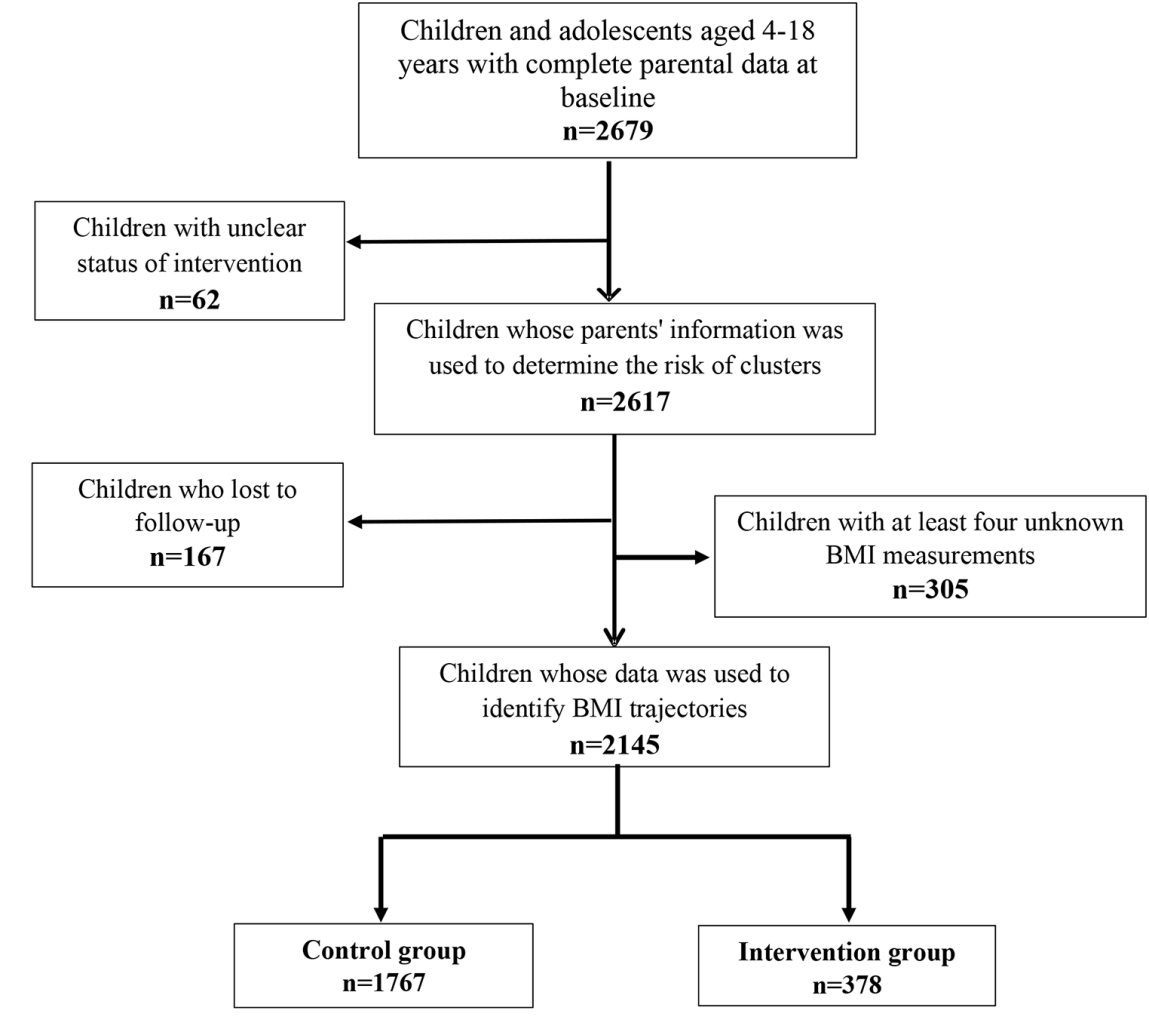
The sampling flowchart

The current study was restricted to participants aged 4–18 years with complete parental data at baseline (n = 2679). After excluding 62 individuals with unknown intervention/control membership, from the remaining 2617 participants, 167 were lost to follow-up during the study (attrition rate was approximately 6%), and 305 participants with at least four unknown BMI were excluded. Subsequently, the BMI trajectories were identified using data of the remaining 2145 participants over the median follow-up of 16.1 years (Fig. 1).

### Measurements

Trained interviewers collected socio-demographic data with standard questionnaires (age, education, occupation, marital status, and medical history). In this regard, characteristics for parents and offspring at baseline and some socio-demographic variables from childhood to young adulthood were considered. Education was categorized as primary, secondary, and higher. Employment status was considered as employed or unemployed. Marital status was categorized as married and unmarried. Blood samples were drawn from all the study participants after overnight fasting of 12–14 h. Parents who met at least three of the following five criteria were considered to have metabolic syndrome (MetS): (1) waist circumference ≥ 90 cm for both sexes; (2) TGs ≥ 150 mg/dL (or antihyperlipidemic medication); (3) HDL-C < 40 mg/dL in males, < 50 mg/dL in females (or antihyperlipidemic medication); (4) SBP ≥ 130 mmHg or DPB ≥ 85 mmHg (or antihypertensive medication); and (5) FPG ≥ 100 mg/dL (or antihyperglycemic medication) [17]. The right arm’s systolic and diastolic blood pressure was measured by a standard mercury sphygmomanometer in a seated position. Measurement was performed twice each visit, with 15 min resting interval. The mean of two measurements was recorded as the participant’s blood pressure. Parental BMI status was defined as normal (< 25 kg/m^2^), overweight (BMI ≥ 25 and BMI < 30), and obese (BMI ≥ 30).

### Trajectory variable

In the current study, BMI was considered the trajectory variable and was calculated as weight in kilograms divided by the height in squared meters (kg/m^2^). Weight was measured using a digital scale with an accuracy of 100 g, with minimal clothing and without shoes. Height was measured using a tape measure in a standing position, without shoes, and with shoulders in a normal position. Waist circumference was measured at the narrowest level with the least clothing [18]. Overweight and obesity were defined using WHO age- and sex-specific BMI cut-offs for children aged 4 to 18 years, averaged across sex at each age [19]. In adults aged ≥ 19 years, BMI ≥ 25 and BMI ≥ 30 were considered overweight and obese, respectively [20].

### Time- stable and time-varying covariates

In the current study, two sets of covariates were considered. (1) Time stable: parental risk cluster and offspring sex at baseline, and (2) Time-varying: intervention status and some individuals’ characteristics which might vary from childhood to young adulthood, including education, marital, and occupation status.

### Parental risk assessment

Considering the importance of parents in developing their children’s weight, the parental risk was assessed using baseline socio-demographic, behavioral, and cardio-metabolic characteristics. Parental risk levels are defined as low and high based on the possible synergic effects of some potentially obesogenic parental characteristics.

### Intervention

The design of the TLGS for lifestyle interventions has been described previously [21, 22]. Briefly, the intervention was adapted from the American Heart Association guidelines and the North Karelia project. The behavioral intervention was inspired by Social Learning Theory tailored for Iranian culture. All participants in the intervention area received a multi-component intervention focusing on three critical aspects of lifestyle, i.e., diet, physical activity, and smoking, with an ultimate goal of controlling NCDs. The professional team of the TLGS trained health volunteers called health liaisons. They worked under the supervision of the intervention health center, recruited participants, and distributed educational materials. The interventions had three components: school-based, family-based, and community-based.

#### School

A large part of the current intervention was implemented in schools. In total, 12 schools in the intervention area were randomly selected as “Health Promoting Schools”; these schools were located at the farthest points from the control area. The TLGS scientific committee trained all principals and volunteer teachers annually. The school-based intervention aimed to modify nutritional habits, physical activity, and smoking and addressed the whole school community (students, teachers, staff, and families). It was delivered by four main methods: (1) classroom curriculum and practice, (2) peer teaching, (3) anti-tobacco policies in schools, and (4) parents’ engagement. The educational content for the classroom curriculum taught students how to lead a healthy lifestyle by targeting their knowledge, attitude, and skills. Nine 45-minute educational sessions were held each year for students in the 7th grade and all new students. An evaluation exam was taken at the end of the year, and the students with low performance participated in the classes again next year. Pamphlets/booklets containing lifestyle education were distributed each year, and students were responsible for taking them to their families. Peer teaching was achieved by forming a “school health society” by the representative students. They worked under the supervision of trained teachers and led other students. Smoking was banned for everyone in school premises. Parents get acquainted with the program by participating in annual two-day seminars followed by 45-minute Q&A sessions. Regular parent-teacher meetings and group discussions were held to help families create a supportive environment for students at home. Further evaluations indicated that almost 70% of planned school-based interventions were successfully implemented.

#### Families

Families were invited for group sessions longer than 2 h, with 10–20 adult participants. Approximately 50% of the intervention population participated in these sessions and received an education, including face-to-face consultation, slide and video presentations regarding healthy food preparation, physical activity benefits, and smoking harms. Through these sessions, smokers were identified and invited to motivational consultation and then referred to cessation clinics. Seasonal health newsletters named “Courier of Health” were distributed to families in the intervention area, which contained health topics (i.e., the food pyramid guide, weight management, health hazards of smoking, smoking-cessation techniques, and the importance of regular physical activity). Pamphlets/booklets were also delivered to all families in the intervention area, two to four times a year. They provided information on food groups, portion sizes, smoking cessation benefits, and coping with stress. Telephone surveys showed that these written material were received and noticed by 50% of households.

#### Community

Community gatherings, including social and religious events, were held two to four times annually to educate the intervention community. Public events were also held in one of the largest local theaters in the intervention area, on occasions such as World No Tobacco Day and World Diabetes Day. Lifestyle-related messages were delivered at these events. Surveys showed that more than 80% of the households participated in at least one of the public gatherings between every two examinations.

Based on the initial objectives of the TLGS and the practical experiences gained, the method of performing the present intervention has changed over time. So, face-to-face and direct training were changed to training by health liaisons (trained volunteers) and written educational media (health courier) at different social levels.

### Statistical analysis

The group-based trajectory models (GBTM) and written program ‘Traj’ (Jones and Nagin, 2007) were used to identify subgroups of participants with similar underlying BMI trajectories between ages four and thirty-six years. Without any priori hypothesis, we investigated the possible number of latent trajectories and a series of models considering several polynomials (cubic, quadratic) and linear specifications of BMI as a function of age. In a GBTM framework, the parameters determining each group’s trajectory are the latent growth factors, i.e., intercepts and slopes. The intercept refers to the initial score at baseline, and the slope corresponds to the rate of change of the trajectory across assessments. Nonlinear trajectories can also be captured by introducing additional quadratic or cubic growth parameters in the model [23]. We began with a single model consisting of one group. We then increased the number of groups until the number of trajectories that best fit the data was identified using the value of the Bayesian Information Criterion (BIC), the average posterior probability of group membership (APP), and the odds of correct classification (OCC). The optimal model was selected as a lower BIC, a higher posterior probability greater than 0.70, and OCC greater than 5. In addition, the size of each class should be at least 5% of our sample [24]. For each subject, the model provides the probability of belonging to each of the identified trajectory groups and assigns the subject to the trajectory group based on the highest chance [23].

A two-step cluster analysis was done to identify parental risk clusters according to the mother and father’s socio-demographic variables, weight status, and metabolic syndrome. The effect of children’s sex and family risk cluster at baseline as time-stable covariates and the intervention status, participant’s marital status, occupation status, and education level at each follow-up as time-varying covariates on the defined trajectory groups were considered in the current GBTM. Estimates for time-stable covariates indicate the increase in the odds ratio of being in a trajectory group (compared to the lowest group) per unit change in the covariate. Estimates for time-varying covariates in each trajectory group indicate how much higher (if the coefficient is positive) or lower (if the coefficient is negative) the BMI trajectory per unit increases in the covariate [24]. We compared children and parental baseline information between control and intervention groups. Continuous variables were expressed as mean ± SD and categorical variables as the frequency (%). The T-test and the Chi-squared tests were used to compare continuous and categorical variables among groups, respectively. All the analyses were conducted in STATA version 16 and IBM SPSS Statistics version 26; two-sided p-values < 0.05 were considered statistically significant.

## Results

The mean age of 2145 participants (49.0% boys) at baseline was 11.52 ± 4.28, and 1767 children were considered the control group. Mean BMI was 18.45 ± 4.37 for both sexes.

### Children and parental characteristics considering control and intervention groups

Table 1 represents the baseline characteristics for children and parents considering intervention and control groups. Among studied characteristics in children, only age was significantly different between control and intervention groups (p < 0.001). In terms of parental features, there were significant differences between age and education levels for both mothers and fathers, BMI status and metabolic syndrome only in mothers, and employment status in fathers between the two groups (p< 0.05). Finally, a significant difference was observed between parental risk clusters in the control and intervention groups.

**Table 1:** Baseline characteristics of children and their parents in intervention and control groups

	Total (n = 2145)	Control (n = 1767)	Intervention (n = 378)	p-value
**Children characteristics**				
**Age (year)**	11.52 ± 4.28	11.33 ± 4.28	12.38 ± 4.13	< 0.001
**Sex** (boy)	1055(49.2)	870(49.2)	185(48.9)	0.955
**BMI (kg/m**^**2**^)	18.45 ± 4.37	18.38 ± 4.43	18.766 ± 4.08	0.122
**Education level**				0.248
Primary	2049 (96)	1694 (96.2)	355 (94.9)	
Secondary	86 (4)	67 (3.8)	19 (5.1)	
**Maternal characteristics**				
**Age (year)**		37.6 ± 7.26	40.169 ± 7.67	< 0.001
**Education**				< 0.001
Illiterate/primary	1165(54.3)	916(51.8)	249(65.9)	
Secondary	849(39.6)	736(41.7)	113(29.9)	
Higher	131(6.1)	115(6.5)	16(4.2)	
**Employment**				0.282
Unemployed	1947(90.8)	1598(90.4)	349(92.3)	
Employed	198(9.2)	169(9.6)	29(7.7)	
**BMI status**				0.033
Normal	553(25.8)	474(26.8)	79(20.9)	
Overweight	965(45.0)	776(43.9)	189(50.0)	
Obese	627(29.2)	517(29.3)	110(29.1)	
**Metabolic syndrome**				0.029
No	1362(63.5)	1141(64.6)	221(58.5)	
Yes	783(36.5)	626(35.4)	157(41.5)	
**Paternal characteristics**				
**Age (year)**		44.09 ± 8.38	47.08 ± 9.01	< 0.001
**Education**				< 0.001
Illiterate/primary	989(46.1)	769(43.5)	220(58.2)	
Secondary	808(37.7)	694(39.3)	114(30.2)	
Higher	348(16.2)	304(17.2)	44(11.6)	
**Employment**				< 0.001
Unemployed	224(10.4)	160(9.1)	64(16.9)	
Employed	1921(89.6)	1607(90.9)	314(83.1)	
**BMI status**				0.261
Normal	779(36.8)	654(37.5)	125(33.8)	
Overweight	990(46.8)	814(46.7)	176(47.6)	
Obese	345(16.4)	276(15.8)	69(18.6)	
**Metabolic syndrome**				0.417
No	1103(53.7)	915(54.1)	188(51.8)	
Yes	950(46.3)	775(45.9)	175(48.2)	
**Parental risk clusters**				< 0.001
High risk	1204(56.1)	944(53.4)	260(68.8)	
Low risk	941(43.9)	823(46.6)	118(31.2)	

Age and BMI are presented as mean ± SD.

Categorical data are presented as numbers (%).

### Parental risk cluster

Figure S1 shows the parental characteristics included in the cluster analysis in order of their importance in differentiating family risk clusters. Maternal education with an importance level of 1.0 is the most influential, and paternal occupation with an importance level of < 0.2 is the latest affecting factor. Based on the distribution of parental characteristics, two distinct clusters were identified and determined as low- and high-risk groups. Smoking and PA were excluded from the model due to low importance values (Table S1).

### Latent BMI trajectories

**Fig. 2:**
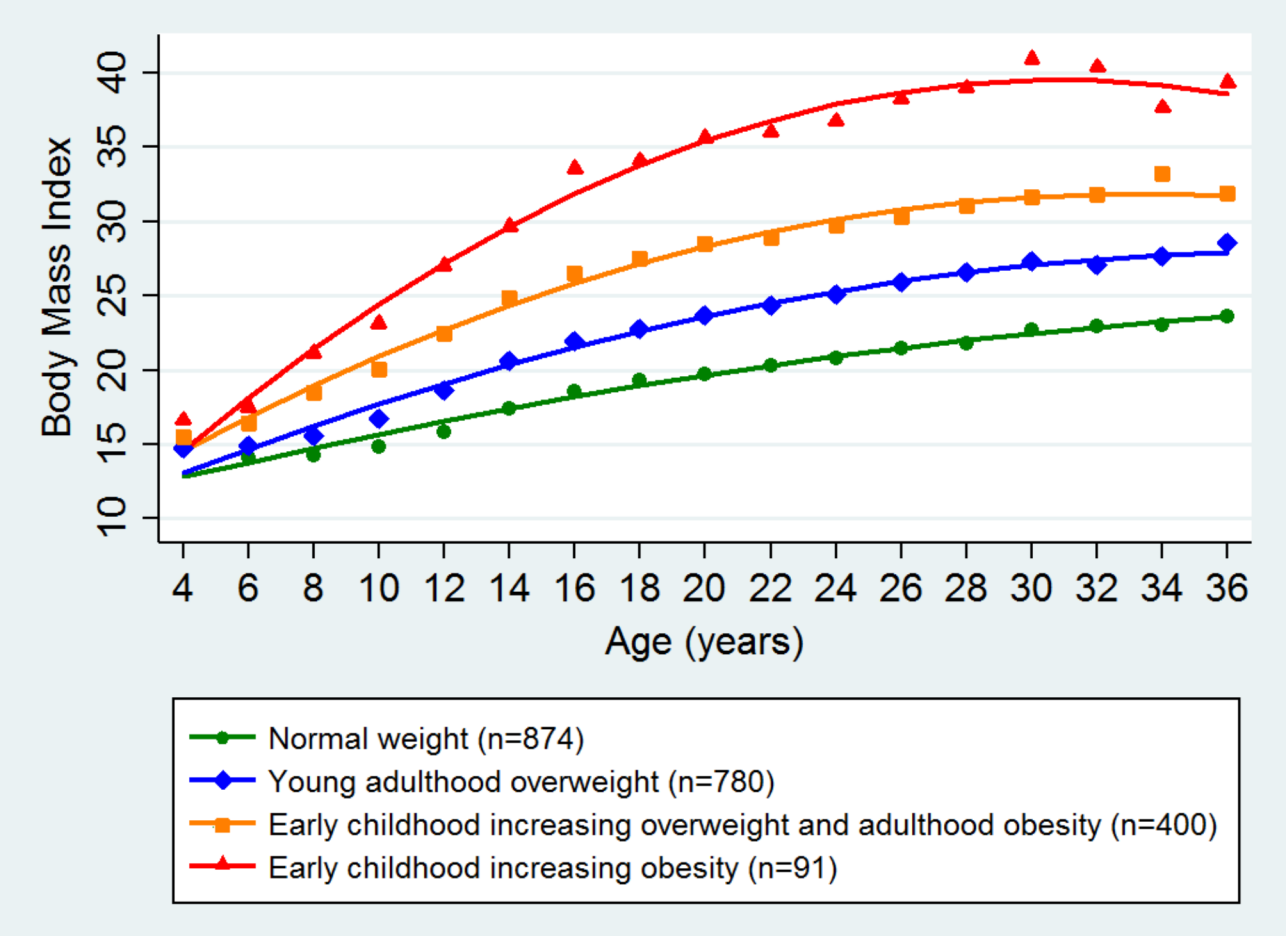
The distinct trajectory patterns of body mass index from childhood (four years) to young adulthood (36 years). The lines show class-specific mean predicted body mass index levels as a function of age estimated from the best fitting group-based model. The green dotted line indicates a stable normal weight group, the blue dotted line indicates early adulthood overweight group, the orange dotted line indicates early childhood increasing overweight group and the red dotted line indicated early childhood obese group. Overweight and obesity were defined using WHO age- and sex-specific BMI cut-offs for children aged 4 to 18 years, averaged across sex at each age. In adults aged 19 years and older, BMI ≥ 25 and BMI ≥ 30 were considered overweight and obese.

Five models with no covariates were fitted to achieve the optimal one. A four-trajectory group model with the quadratic term was favored based on reduction in BIC (Table S2). Each group’s average posterior probability value in the four-group model ranged from 0.89 to 0.96. The estimated group sizes were comparable with the actual group sizes, suggesting a good fit. The odds of correct classification exceeded 5, suggesting accurate group assignment. Since this model was the most parsimonious one, it has been utilized for categorizing BMI changes from childhood (4 to 18 years) to early adulthood (22 to 36 years). Four trajectory groups of BMI were identified in all participants. They were labeled as normal weight (NW), young adulthood overweight (YAO), early childhood increasing overweight and adulthood obesity (ECOAO), and early childhood increasing obesity (ECO). In total, 874 individuals followed a normal weight trajectory with a normal average predicted BMI levels throughout the study period. Participants following the young adulthood overweight trajectory (n = 780) experienced a gradual increase in BMI starting from early adulthood and remaining in the range of overweight (BMI ≤ 30) through the follow-ups. The participants in the early childhood increasing overweight and adulthood obesity (n = 400) followed a trajectory of increasing BMI that led to obesity in early adulthood. The participants in the early childhood increasing obesity group (n = 91) were obese from early childhood. They followed a trajectory of growing obesity up to a BMI of 40 throughout their observed life course (Fig. 2).

### Time-stable and time-varying predictors of BMI trajectories

Table 2 presents the impact of time-stable covariates. As results showed, compared to males, females had lower odds of being in the YAO, ECOAO, and ECO groups rather than the NW group (OR = 0.70, 0.48, and 0.64, respectively). However, compared to the NW trajectory group, there was no significant difference between the parental risk levels among the other three trajectory patterns of BMI over time.

**Table 2:** Time-stable predictors of BMI trajectory groups based on the offspring sex and parental risk clusters

		Odds ratio	SE	p-value
**Female (**vs. **Male)**				
	Normal weight	1		
	Young adulthood overweight	0.70	0.12	0.04
	Early childhood increasing overweight and adulthood obesity	0.48	0.13	< 0.001
	Early childhood increasing obesity	0.64	0.23	0.051
**High risk (**vs. **Low risk)**				
	Normal weight	1		
	Young adulthood overweight	0.001	0.12	0.990
	Early childhood increasing overweight and adulthood obesity	1.13	0.13	0.347
	Early childhood increasing obesity	1.21	0.23	0.397

Odds of being in each BMI trajectory group compared to the stable normal group were reported. Models adjusted for intervention status, marital status, occupation status, and education level as time-varying covariates, and sex and family risk cluster at baseline as time-stable covariates.

Table 3 shows the impacts of time-varying covariates. Our results showed that YAO and ECO trajectories in the intervention group were concomitant with lower BMI levels than in the control group, with the highest estimated effect in the latter (β=-0.52 and p = 0.018; β= -1.48 and p < 0.001, respectively). In addition, the current results indicated that being unmarried was associated with a lower BMI in all trajectory groups (β values ranged from − 1.27 for ECO to -0.68 for NW, p < 0.01). Similarly, higher levels of education were associated with lower BMI in all trajectory groups (β values ranged from − 1.35 for ECO to -0.28 for YAO, p < 0.05). Unemployment was related to lower BMI in YAO (β=-0.47 and p < 0.001) and ECOAO trajectory groups (β=-0.51 and p = 0.007).

**Table 3:** Body mass index trajectory groups and the effects of time varying predictors on their shape

		Estimate	SE	p-value
**Normal weight (NW)**				
	Intercept	13.33	1.13	< 0.001
	Linear	0.53	0.09	< 0.001
	Quadratic	-0.01	0.00	0.002
**Time varying covariates**				
	Intervention status	-0.11	0.15	0.481
	Marital status	-0.68	0.13	**< 0.001**
	Occupation status	-0.67	0.12	0.0580
	Education level	-0.34	0.11	**0.002**
**Young adulthood overweight (YAO)**				
	Intercept	15.12	1.20	< 0.001
	Linear	0.82	0.01	< 0.001
	Quadratic	-0.01	0.00	< 0.001
**Time varying covariates**				
	Intervention status	-0.52	0.22	**0.018**
	Marital status	-1.23	0.15	**< 0.001**
	Occupation status	-0.47	0.13	**< 0.001**
	Education level	-0.28	0.12	**0.022**
**Early childhood increasing overweight and adulthood obesity (ECOAO)**				
	Intercept	19.29	1.76	< 0.001
	Linear	0.90	0.14	< 0.001
	Quadratic	-0.01	0.00	< 0.001
**Time varying covariates**				
	Intervention status	-0.40	0.27	0.134
	Marital status	-1.22	0.21	**< 0.001**
	Occupation status	-0.51	0.19	**0.007**
	Education level	-0.59	0.16	**< 0.001**
**Early childhood increasing obesity (ECO)**				
	Intercept	19.31	3.67	< 0.001
	Linear	1.59	0.30	< 0.001
	Quadratic	-0.02	0.00	< 0.001
**Time varying covariates**				
	Intervention status	-1.48	0.40	**< 0.001**
	Marital status	-1.27	0.46	**0.006**
	Occupation status	-0.64	0.39	0.095
	Education level	-1.35	0.34	**< 0.001**

Models adjusted for intervention status, marital status, occupation status and education level as time-varying covariates, and sex and family risk cluster at baseline as time-stable covariates.

†In each group, estimates represent the shift in BMI status per unit change in exposure variables (intervention vs. control, unmarried vs. married, unemployed vs. employed, high levels education vs. low levels).

## Discussion

This study examined the effect of multi-setting healthy lifestyle intervention on long-term BMI trajectories in children and adolescents who participated in the TLGS from 1998 to 2018. Our results provide evidence of four developmental trajectories of BMI, from childhood to young adulthood. Most participants followed a trajectory in which the average BMI levels remained within the normal range throughout the study period. Boys were more likely to be categorized in vulnerable BMI trajectory groups. BMI status has been significantly affected by changes in marital status, occupation, and education levels over time. Regarding healthy lifestyle education, the present results showed that compared to the control group, significant BMI reduction was observed in those who had experienced a growing trend of obesity from early childhood and those whose onset of excessive weight gain was in youth.

Previous studies have examined BMI change patterns during different periods of life. All the trajectory groups showed an increasing trend during young adulthood, but with different baseline levels and incremental gradients in the current study [25–29]. Most of our participants followed a normal BMI in line with previous studies. Although the second large subpopulation identified in our analysis was normal weight in childhood, it transitioned to overweight in young adulthood. In the previous study on the TLGS population, three BMI trajectories were identified as normal, overweight, and increasing overweight/obese [30]. However, the current study allowed us to differentiate two subgroups (ECOAO and ECO) from the previous increasing overweight/obese group with a larger sample size. The former experienced overweight before 18, which led to obesity in adulthood. The latter is a small group whose BMI started increasing from mid-adolescence with the largest slope. They remained obese until the end of the follow-up period and experienced BMIs of about 40. Such trajectories with excessive and rapid weight gain were detected in other populations, but they had different and mostly sooner onset of overweight/obesity than our population [26]. In addition to the stable and rising trajectories, some previous studies captured decreasing transition from being overweight/obese to normal [31].

Our results show that the female sex was associated with lower odds of following higher BMI trajectories. In line with previous studies findings [32, 33], higher education level and singlehood were associated with lower BMIs in all trajectories. Evidence suggests that educated people engage in more PA, belong to higher socio-economic positions, and access healthier foods [32, 34]. Moreover, it is hypothesized that married individuals are out of the “marriage market”, therefore pay less attention to their body shape and consume more dense food [35]. Our results showed that unemployment reduced BMI only in YAO and ECOAO groups regarding the job status. It has been argued that employed individuals may have less leisure time to engage in PA; thus, most of their PA is work-related, which has less impact on weight and other cardiovascular risk factors.

Among numerous implemented programs focusing on improving children’s weight status, multicomponent lifestyle interventions are commonly used and considered the preferred methods to prevent and treat childhood overweight [36, 37]. Our findings expand existing evidence as it is the first to investigate such educational intervention on long-term BMI trajectories; however, comparing obtained results with similar interventions is not possible. The current intervention reduced the incremental slope of BMI in two of the at-risk trajectories, i.e., YAO and ECO. Possible hypotheses in the YAO group could be a better perception of the health-related threats posed by excessive weight gain, negative body image, and its social consequences. In addition, children who were obese from adolescence benefited more from the educational intervention than normal or overweight children. It could be because of the higher threat perceptions among parents of the former group. In Eastern Mediterranean countries such as Iran, the society has an overall positive view of childhood overweight and considers it more a sign of well-being, beauty, and wealth than a threat to health [38]. On the other hand, factors such as selling unhealthy foods in school neighborhoods, a built environment unsupportive of PA, child-directed fast-food commercials, the high cost of healthy foods, and educational priorities in adolescents could act as barriers to lifestyle change [39].

This study has some limitations that should be considered, including the non-randomized design of the TLGS, which was inevitable to increase community outreach and feasibility at the population level. On the other hand, any talk about a healthy lifestyle between individuals from intervention and control areas may mask the effect of lifestyle education. Because it is a broad community trial, it is impossible to determine the impact of each part of the intervention separately. Finally, lifestyle behaviors were assessed based on the participants’ self-reports using relevant questionnaires, which could raise recall bias. The current study is the first to investigate the effectiveness of a practical multi-setting healthy lifestyle intervention on developmental BMI trajectories in the Middle East. The large sample size and long-term follow-up of more than 18 years are among the strengths of this study.

## Conclusion

According to our results, long-term multi-setting lifestyle intervention reduced BMI in individuals who were persistently obese from early adolescence and normal-weight children who became overweight in youth. Current results provide preliminary knowledge for designing more targeted and efficient weight-related public health interventions that can be translated into policy. Our findings yield evidence to help policymakers benefit from low-cost lifestyle interventions in BMI reduction and encourage them to avoid a one-size-fits-all approach to obesity control. Other underlying factors should be considered in community-wide weight management programs, including but not limited to readiness levels, social beliefs towards obesity, and threat perception both from children and their parents.

## Electronic supplementary material

Below is the link to the electronic supplementary material.

Supplementary Material 1

## Data Availability

The datasets used and/or analyzed during the current study are available from the corresponding author on reasonable request.

## Abbreviations

TLGS: Tehran lipid and glucose study.
BMI: Body mass index.
NCD: Non-communicable disease.
WHO: World health organization.
GBTM: Group based trajectory model.
APP: Average posterior probability.
BIC: Schwartz Bayesian criterion.
OCC: Odds of correct classification.
PA: Physical activity.
NW: Normal weight.
YAO: Young adulthood overweight.
ECOAO: Early childhood increasing overweight and adulthood obesity .
ECO: Early childhood increasing obesity.
