# Supplementary material for: Effectiveness of a practical multi-setting lifestyle intervention on the main BMI trajectories from childhood to young adulthood: A community-based trial
Source: BMC Public Health. 2022 Oct 31;22:1995. doi: 10.1186/s12889-022-14306-2 (PMC9624045; doi:10.1186/s12889-022-14306-2)
Supplement: Supplementary file 1 — Supplementary Material 1 [file 12889_2022_14306_MOESM1_ESM.docx]

**Table S1.** Parental characteristics based on the low and high-risk clusters

|  | | **Low risk**  **(n=941)** | **High risk**  **(n=1204)** | **P-value** |
| --- | --- | --- | --- | --- |
| **Maternal characteristics** | |  |  |  |
|  | **Age** | 35.39 (5.78) | 40.12(7.85) | <0.001 |
|  | **Education** |  |  | <0.001 |
|  | Illiterate/primary | 155 (16.5) | 1010 (83.9) |  |
|  | Secondary | 657 (69.8) | 192 (15.9) |  |
|  | Higher | 129 (13.7) | 2 (0.2) |  |
|  | **Employment status** |  |  | <0.001 |
|  | Unemployed | 743 (79.0) | 1204 (100.0) |  |
|  | Employed | 198 (21.0) | 0 (0.0) |  |
|  | **BMI status** |  |  | <0.001 |
|  | Normal | 363 (38.6) | 190 (15.8) |  |
|  | Overweight | 473 (50.3) | 492 (40.9) |  |
|  | Obese | 105 (11.2) | 522 (43.4) |  |
|  | **Metabolic syn.** |  |  | <0.001 |
|  | No | 827 (87.9) | 535 (44.4) |  |
|  | Yes | 114 (12.1) | 669 (55.6) |  |
| **Paternal characteristics** | |  |  |  |
|  | **Age** | 40.97 (6.17) | 47.47 (9.08) | <0.001 |
|  | **Education** |  |  | <0.001 |
|  | Illiterate/primary | 126 (13.4) | 863 (71.7) |  |
|  | Secondary | 570 (60.6) | 238 (19.8) |  |
|  | Higher | 245 (26.0) | 103 (8.6) |  |
|  | **Employment status** |  |  | <0.001 |
|  | Unemployed | 28 (3.0) | 196 (16.3) |  |
|  | Employed | 913 (97.0) | 1008 (83.7) |  |
|  | **BMI status** |  |  | 0.005 |
|  | Normal | 344 (37.1) | 435 (36.7) |  |
|  | Overweight | 459 (49.5) | 531 (44.8) |  |
|  | Obese | 125 (13.5) | 220 (18.5) |  |
|  | **Metabolic syn.** |  |  | 0.004 |
|  | No | 512 (57.3) | 591 (50.9) |  |
|  | Yes | 381 (42.7) | 569 (49.1) |  |

**Table S2.** Group Based models results.

| **Nb.**  **Latent**  **classes** | **Polynomial degree** | **Log-Lik** | **BIC** | **% Participants per class** | **Mean posterior**  **probabilities** | **Posterior probabilities** | **odds of correct classification** |
| --- | --- | --- | --- | --- | --- | --- | --- |
| **1** | Linear | -29702.09 | -29713.59 | 100 | NA | NA | NA |
|  | Quadratic | -29573.00 | -29588.34 | 100 | NA | NA | NA |
|  | Cubic | -29572.60 | -29591.78 | 100 | NA | NA | NA |
| **2** | Linear | -27452.58 | -27475.60 | 71.40 , 28.60 | 0.97 , 0.95 | 71.4 , 28.6 | 15.23 , 43.54 |
|  | Quadratic | -27125.31 | -27155.99 | 71.12 , 28.87 | 0.98 , 0.95 | 71.1 , 28.9 | 19.31 , 42.13 |
|  | Cubic | -27117.17 | -27155.52 | 71.18 , 28.82 | 0.98 , 0.95 | 71.2 , 28.8 | 18.20 , 45.39 |
| **3** | Linear | -26527.57 | -26562.09 | 55.83 , 36.06 , 8.11 | 0.95 , 0.92 , 0.95 | 55.8 , 36.1 , 8.1 | 15.5 , 20.04 , 210.95 |
|  | Quadratic | -26076.89 | -26122.91 | 55.71 , 36.30 , 7.99 | 0.96 , 0.93 , 0.95 | 55.7 , 36.3 , 8.0 | 17.33 , 25.63 , 209.97 |
|  | Cubic | -26069.80 | -26127.33 | 55.71 , 36.27 , 8.02 | 0.96 , 0.93 , 0.95 | 56.1 , 35.8 , 8.01 | 17.51 , 25.21 , 218.05 |
| **4** | Linear | -26090.78 | -26136.80 | 40.22 , 36.74 , 18.47 , 4.56 | 0.91 , 0.88 , 0.92 , 0.96 | 40.2 , 36.7 , 18.5 , 4.6 | 15.54 , 12.71 , 50.81 , 485.63 |
|  | **Quadratic** | **-25526.03** | **-25587.40** | **40.69 , 36.33 , 18.73 , 4.26** | **0.93 , 0.89 , 0.93 , 0.96** | **40.7 , 36.4 , 18.6 , 4.2** | **21.11 , 14.94 , 56.08 , 523.99** |
|  | Cubic | -25512.87 | -25589.58 | 36.43 , 40.53 , 18.73 , 4.30 | 0.89 , 0.94 , 0.93 , 0.96 | 36.4 , 40.5 , 18.7 , 4.3 | 14.40 , 22.08 , 56.48 , 506.40 |
| **5** | Linear | -25953.52 | -26011.05 | 37.23, 36.55, 19.31, 5.86, 1.03 | 0.90, 0.87, 0.89, 0.91, 0.98 | 37.2, 36.5, 19.3, 5.8, 1.0 | 14.91, 12.10, 35.35, 172.80, 4404.99 |
|  | Quadratic | -25325.61 | -25402.32 | 40.57, 36.30, 4.25, 18.73, 0.14 | 0.94, 0.90, 0.97, 0.93, 1 | 40.6, 36.3, 4.3, 18.7, 0.1 | 22.43, 15.56, 649.10, 58.16, NA |
|  | Cubic | -25317.58 | -25413.46 | 36.24, 40.25, 17.71, 1.48, 4.31 | 0.90, 0.94, 0.92, 0.83, 0.96 | 36.2, 40.3, 17.7, 1.5, 4.3 | 15.04, 23.17, 54.75, 379.10, 581.48 |

Reported are: The number of latent class considered, the polynomial form of the model, the maximum Log-Likelihood (Log-Lik), the Bayesian information Criterion (BIC), and for models with 2 or more classes, the a-posteriori classification of subjects in each class (%), the mean of posterior probabilities in each latent class, and the % of subjects classified in each class with a posterior probability above 0.7. The best fitting model is highlighted in bold characters. (n.a: not applicable).


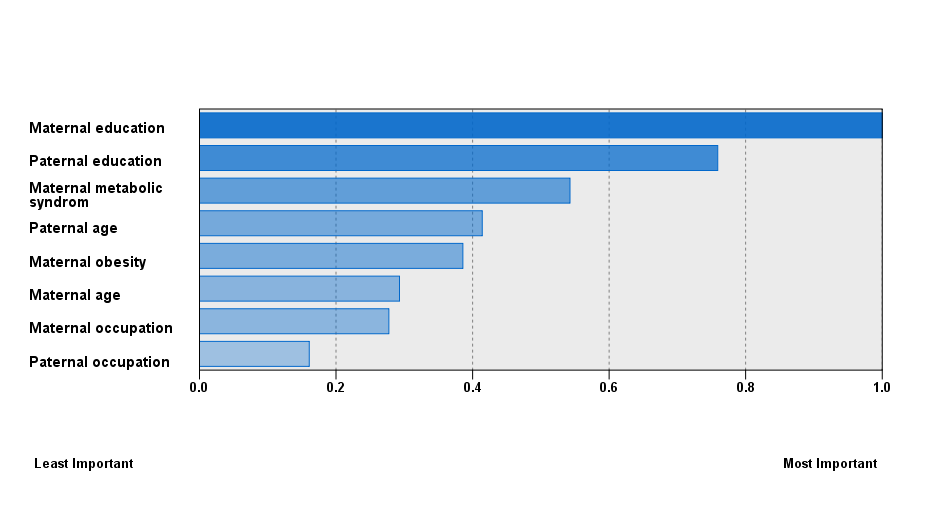


**Figure S1.** Importance values of parental characteristics included in the cluster analysis
